# Acceptance of team-based learning by students and faculty: A pilot study

**DOI:** 10.12669/pjms.40.5.8515

**Published:** 2024

**Authors:** Rehana Rehman, Saara Ahmad, Syeda Pinar Nasir, Rahila Ali

**Affiliations:** 1 Rehana Rehman, Professor, Department of Biological & Biomedical Sciences. Aga Khan University, Karachi - Pakistan; 2 Saara Ahmad, Assistant Professor, Department of Biological & Biomedical Sciences. Aga Khan University, Karachi - Pakistan; 3 Syeda Pinar Nasir, Department of Biological & Biomedical Sciences. Aga Khan University, Karachi - Pakistan; 4 Rahila Ali, Senior Instructor, Department for Educational Development. Aga Khan University, Karachi - Pakistan

**Keywords:** Team Based Learning, Students engagement, Self-accountability

## Abstract

**Background & Objective:**

Team-Based Learning (TBL) is an interactive instructional approach characterized by collaborative peer teaching in both large and small group settings. The study aims to assess usefulness of the TBL in enhancing student learning outcomes and engagement in graduate classes.

**Methods:**

This mixed method study was conducted from January 2023 till July 2023 at the Department of Biological & Biomedical Sciences at Aga Khan University, Karachi, Pakistan, a questionnaire was distributed to graduate students in Endocrine and Reproductive course after TBL on ‘Hormonal changes in Pregnancy’. Focus group discussion (FGD) was held with facilitator of this TBL and the students; results of both arms were then triangulated.

**Results:**

All (four) students responded affirmatively regarding guided self-preparation, quality of application exercises, satisfaction in terms of student’s engagement, a positive attitude and self-accountability. Themes identified by FGD of both students and facilitators were ‘Students Engagement in Peer Learning, ‘Conducive Learning Environment’, “Time is Capital in TBL’ and ‘Conceptual learning.’

**Conclusion:**

The pilot study confirmed the utility of TBL by students as well as the facilitators. Students came with prior preparation, got engaged in problem-solving activities and received feedback from peers and the expert facilitators. The conducive environment enhanced their engagement, enabled them to actively apply the content and benefit from guided supervision.

## INTRODUCTION

As economic and technological advancements in medical knowledge and scientific research continue to evolve, globalization is increasingly gaining importance in the field of medical education.^1^ One pedagogical strategy, known as team-based learning (TBL), effectively involves students by combining individual testing with collaborative group work.^2^ TBL has been identified as best practice of evidence-based teaching involving active instructional strategy of small groups to improve student engagement, promote deep understanding of concepts and facilitate team working skills.^3^,^4^ The students apply their knowledge interactively which encourages individuals to collaborate in teams and develop critical thinking abilities.^5^ TBL has been implemented as a preferred mode of learning in number of medical and health schools.^6^,^7^

TBL process is carried out through following steps; I) Advance Assignment, guided self-preparation with pre-reading materials for initial self-directed acquisition of knowledge. II) an Individual Readiness Assurance Test (iRAT) to assess the basic understanding of data and concepts learnt through Step-I which will help to solve Team Application (tAPP) problems. The same test is then discussed within groups of 5-7 learners [Group Readiness Assurance Test (GRAT)] in which teams receive immediate feedback by the faculty and indulge in discussions to clarify questions through team discussion as well as from the facilitators. In Step-III, students apply what they have learnt during the first II steps to analyze real world problems.^8^ In the final steps, students are given the chance to appeal and provide peer evaluation to the facilitators. With the help of these steps an effective positive learning environment is created which saves time, facilitates construction of knowledge, critical thinking, and improves academic performance.^9^,^10^

We are looking forward to a large-scale implementation of TBL at AKU in graduate classes but before that we have piloted “TBL” in one of the graduate classes, exploring perception of students and facilitators regarding the teaching method. The study is therefore aimed to assess usefulness of team-based learning strategy through this study involving students and facilitators.

## METHODS

A mixed method study was conducted at the department of Biological & Biomedical Sciences at Aga Khan University, Karachi, Pakistan from January 2023 till July 2023. comprising of quantitative aspect through evaluation of responses by a validated questionnaire and qualitative by Focus group discussions (FGD) with all (four) students and facilitators (two) who conducted the TBL

### Ethical Approval

The study was approved by the institutional Ethics Committee Ref. (ERC-2023-8206-23887)

### Description of Questionnaire

The questionnaire comprised of six major sub scales; Pre-reading, Readiness Assurance Test, Application Exercises, learning during TBL, Preferences on other teaching methodologies, Self-Accountability and Student satisfaction. These major subscales were further divided into a total of 55 items/ scored from a range of 0-6, (0 being the least satisfactory, three being the neutral and 6 being the most satisfactory). The questionnaire was administered to at least five graduate students to validate the instrument. The reliability coefficient was 0.8. Construct validity was acquired by literature search (books and manuals), content validity was ensured by aligning with the objectives and obtaining feedback from three content experts from disciplines of Physiology, Gynaecology and Medical Education. Students were informed about the process; asked to respond by rating their level of satisfaction to each item in the subscale. All the students (four) were included. There are no definite exclusion criteria.

### Description of FGD

A semi-structured interview guide for the facilitators and students was developed, reviewed and piloted before the discussions. One FGD was conducted with the students (n=4) and one with facilitators (n=2). Both FGDs were conducted for a period of 45 to 60 minutes, transcribed, member checked and from the extracted data; codes, categories and themes were identified.

### Data Analysis

Quantitative data was analyzed using SPSS Version 20 for descriptive analysis of students satisfaction to the new methodology; frequencies and percentages were calculated. The transcriptions of the Focus Group Discussions (FGDs) were carefully examined for thematic analysis, codes, and themes were identified. Identity of participants remained anonymous, and data gathered from focus group discussion and student evaluation remained confidential.

### Results of Quantitative Analysis

Students satisfaction was determined through the questionnaire and an average score of the items in each subscale was taken. The mean score and standard Deviation (SD) of all scales is illustrated in Table-I. The survey results as shown in Table-II conclude that the average satisfaction level is high (*x̅* = 6.0) in the construct “Student Satisfaction” while it is low and considerable (*x̅* = 4.8) in the construct “Pre-reading, Readiness Assurance Test” which is measured on five-point Likert scale. Similarly, the variation is high (SD = 1.9) in the “Pre-reading, Readiness Assurance Test” while it’s minimum (SD = 0.2) in “Student Satisfaction”.

**Table-I:** Mean Satisfaction Level with Dispersion.

Items	Mean	SD
***Pre-reading, Readiness Assurance Test***		
Placement in schedule was appropriate	1.5	1.0
Time allocated was adequate	4.5	1.7
Schedule given well before time	4.0	2.0
Expected outcomes outlined	5.8	0.5
Working groups pre-defined	4.5	1.9
Adequate access to sufficient resources provided	6.0	0.0
Readiness Assurance Tests assessed depth of knowledge	4.0	2.8
Individual Readiness Assurance Test (IRAT) recollected concepts	6.0	0.0
Group Readiness Assurance Test (GRATS) enabled information recall	6.0	0.0
Enabled to rationalize the problem after GRAT	6.0	0.0
***Application Exercises***		
Applied linking of concepts	6.0	0.0
Aided retention of concepts	5.8	0.5
Assisted knowledge sharing	5.8	0.5
Facilitated peer participation	5.8	0.5
Empowered to communicate effectively	6.0	0.0
Fostered team communication skills	5.5	0.6
Improved patience to listen to comments	5.5	1.0
Stimulated problem-solving skills	5.8	0.5
Contributed to leadership skills	5.3	1.0
***Learning during TBL***		
Created a supportive learning environment	6.0	0.0
Consolidated concepts easily	5.5	0.6
Encouraged active participation in discussions	6.0	0.0
Facilitated learning from peer feed back	5.8	0.5
Aligned concentration with the instructor	5.8	0.5
Facilitated learning from tutor feed back	5.5	1.0
Promoted analytical thinking	5.8	0.5
Developed clinical reasoning skills	5.8	0.5
Respected everyone’s opinion	6.0	0.0
***References on other teaching methodologies***		
Focused concentration in TBL as compared to lectures	6.0	0.0
Enhanced ability to search information more than lectures	6.0	0.0
Improved the ability to speak more than lectures	4.5	3.0
Worked hard to meet lecturers’ expectations more than lectures	5.0	2.0
Improved recall in examination more than lectures	6.0	0.0
Focused concentration in TBL as compared to PBL	5.8	0.5
Improved collaborative learning as compared to PBL	6.0	0.0
Enhanced understanding of concept(s) more than PBL	6.0	0.0
Facilitated learning in small group more than PBL	6.0	0.0
Effective completion of task by TBL more than PBL	6.0	0.0
Improved recall in examination more than PBL	6.0	0.0
***Self-Accountability***		
Motivated to come prepared	6.0	0.0
Came fully prepared for the class	6.0	0.0
Felt accountability for learning	5.5	0.6
Dedicated more time in team activities	6.0	0.0
Exhibited accountability in teamwork	6.0	0.0
Extended support to team members	6.0	0.0
Felt proud to assist team members	6.0	0.0
***Student satisfaction***		
Enjoyed learning activities	6.0	0.0
Enhanced student’s engagement	6.0	0.0
Received constructive criticism	5.8	0.5
Felt valued to share educational experiences	6.0	0.0
Felt respected	6.0	0.0
Felt pride of my work	6.0	0.0
Developed positive attitude	6.0	0.0
Experienced it as an effective approach	6.0	0.0
Interested to participate in similar activities	6.0	0.0

**Table-II:** Overall Average Satisfaction and Variation among the Constructs.

Construct	Mean	SD
Pre-reading, Readiness Assurance Test	4.8	1.9
Application Exercises	5.7	0.6
Learning during TBL	5.8	0.5
References on other teaching methodologies	5.8	1.1
Self-Accountability	5.9	0.3
Student satisfaction	6.0	0.2

### Results of Qualitative Analysis

Following themes emerged on account of FGD with facilitators and students.

### Students Engagement and Peer Learning

Students mentioned that TBL promoted active participation through individual quizzes (iRAT) and collaborative problem solving (GRAT), these contributed to improved understanding. A student replied; “we were able to score better in GRAT and were able to clarify some concepts from our peers”. Furthermore, one of the facilitators responded, “TBL encouraged students to engage in peer-to-peer teaching and learning”

**Fig.1:**
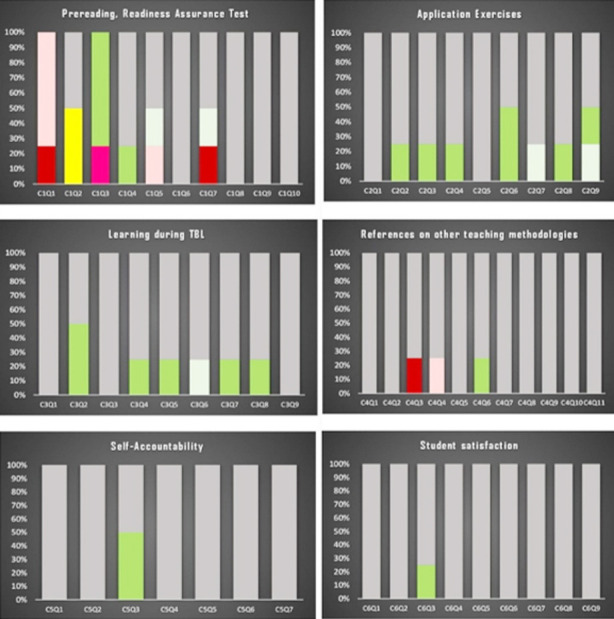
provides an insight into the overall perception of TBL and indicates the degree of satisfaction experienced by the participants for each item.

### Conducive Learning Environment

A facilitator responded, “--I found TBL to be an engaging and interactive pedagogical approach that encourages collaboration, critical thinking, and ownership of learning. The use of RATs, team discussions, and immediate feedback all contribute to an effective and dynamic learning environment that can lead to improved student outcomes”. A student mentioned “Having access to learning resources before the session boosted our confidence in preparedness for the session.” This can foster a positive learning environment and encourage students to actively engage in the learning process.

### Time is Capital in TBL

Students found reading resources valuable and were particularly satisfied with the usefulness of videos, which effectively clarified their concepts. One of the students stated, “we were given a good platform with healthy reading material which helped in clarification of concepts”. The facilitator mentioned: “Despite the potential time-consuming nature of planning for TBL, I have found that the investment of time and effort is worthwhile.”

### Conceptual learning

“Overall, as a facilitator, I find TBL to be an engaging and interactive pedagogical approach that encourages collaboration, critical thinking, and ownership of learning.”

### Triangulation of Results

The results of scores from the questionnaire and thematic analysis highlighted usefulness of TBL as an effective teaching tool that enhanced student’s engagement, teamwork and responsiveness to learning.

## DISCUSSION

Our study identified themes of student engagement and peer learning and facilitators acknowledged usefulness of TBL for enhancing engagement, teamwork skills, deeper learning, and creating a supportive learning environment. TBL offers active learning through relevant problems and group interaction. This fosters students’ student’s engagement and reinforces teamwork skills by encouraging critical analysis during problem solving exercises and resolving conflict during the group discussions. A study also supported these results indicating that students experienced engagement through team support and trust for learning.^11^ Importance of online TBL sessions in students learning has been documented.^12^

In another study comparing TBL with PBL, students preferred TBL as a conducive learning approach encouraging active participation with deeper engagement in the learning process and mastery of course content.^13^ The usefulness of TBL is proved over other teaching methodologies.^14^-^16^ Generally, students are satisfied with TBL, and student engagement is also higher in TBL classes. Another theme that emerged in our FGD was conceptual learning and this is reinforced in other studies as well.

In a study conducted in Pakistan; student achieved higher mean scores by virtue of content taught in TBL.^17^ Our study participants reinforced the usefulness of TBL in promoting student engagement and learning outcomes. This is supported by improvement in students’ overall learning satisfaction through substantial engagement, captivating and smooth interaction, active participation, enhanced presentation skills, and proficiency in effective communication.^18^ Learners’ involvement and social interaction during the teamwork observed in our study is supported by the literature.^19^,^20^ TBL presents a promising alternative to traditional didactic methods, with the potential to enhance students learning efficiency. It does so by implementing a structured learning cycle that emphasizes accountability within a collaborative learning team.^21^,^22^ TBL has also proved to increase knowledge and student’s satisfaction in bioethics curriculum of residency students.^21^

Students mentioned the utility of pre-reading material. Literature also supports that TBL shifts the burden of learning content during class with the pre-reading material.^22^ Graduate students appreciated an active and participatory learning environment during classroom activities in particular the RATs application exercises and timely feedback which corroborates with literature.^23^ They highlighted importance of RAT for comprehension and rationalization of the session content. The immediate feedback provided during RATs allowed them to reflect on their performance and actively engage in discussions with their teams to resolve any discrepancies or uncertainties.^24^ During the TBL when students solve clinical problem-solving activities, they use their shared knowledge, clinical reasoning skills, ethical views and values which enable them to know and solve complex clinical problems in real life situation.

### Limitations

Our study’s limitations include a small sample size and being conducted in a single session, which may not fully capture the long-term effects of implementing TBL. Although TBL has been known to improve learning and performance, this study did not assess exam performance. Nevertheless, this pioneer study lays the groundwork for introducing TBL in graduate classes for further implementation. The findings from this study will contribute to the existing body of knowledge on pedagogical methodologies and serve as a basis for informed decision-making by educators and institutions seeking evidence-based approaches to optimize graduate-level teaching and learning. Ultimately, the study endeavors to advance the scholarship of teaching and learning, fostering effective educational practices that empower students to reach their full academic potential.

## CONCLUSION

The pilot study confirmed that TBL is effective for both students and facilitators. Students came prepared, actively engaged in problem-solving, received feedback from peers and expert facilitators, and benefited from a conducive learning environment that encouraged active application and guided supervision.

### Authors Contribution:

**RR:** Principal investigator, designed the study, supervised the whole project reviewed the manuscript.

**SM:** Assisted writing the manuscript.

**SPN:** Assisted in data analysis.

**RA:** Conducted the FGD and assisted in data analysis, formatted the write up as per guidelines of the journal and responsible for accuracy of the study.

All authors have read the manuscript.
